# Contrasting Roles of E2F2 and E2F3 in Cardiac Neovascularization

**DOI:** 10.1371/journal.pone.0065755

**Published:** 2013-06-14

**Authors:** Junlan Zhou, Min Wu, Shiyue Xu, Min Cheng, Caizhi Ding, Ye Liu, Hongbin Yan, Dauren Biyashev, Raj Kishore, Gangjian Qin

**Affiliations:** 1 Feinberg Cardiovascular Research Institute, Department of Medicine – Cardiology, Northwestern University Feinberg School of Medicine, Chicago, Illinois, United States of America; 2 Department of Plastic Surgery, Tongji Hospital, Huazhong University of Science and Technology, Wuhan, Hubei, P. R. China; 3 Department of Cardiology, Union Hospital, Tongji Medical College, Huazhong University of Science and Technology, Wuhan, Hubei, P. R. China; 4 Department of Cardiology, Renmin Hospital, Songzi, Hubei, P. R. China; 5 Laboratory, Renmin Hospital, Songzi, Hubei, P. R. China; 6 Cardiology Department, Cardiovascular Institute and Fuwai Hospital, Chinese Academy of Medical Sciences and Peking Union Medical College, Beijing, P. R. China; University of Cincinnati, United States of America

## Abstract

Insufficient neovascularization, characterized by poor endothelial cell (EC) growth, contributes to the pathogenesis of ischemic heart disease and limits cardiac tissue preservation and regeneration. The E2F family of transcription factors are critical regulators of the genes responsible for cell-cycle progression and growth; however, the specific roles of individual E2Fs in ECs are not well understood. Here we investigated the roles of E2F2 and E2F3 in EC growth, angiogenesis, and their functional impact on myocardial infarction (MI). An endothelial-specific E2F3-deficient mouse strain VE-Cre; E2F3^fl/fl^ was generated, and MI was surgically induced in VE-Cre; E2F3^fl/fl^ and E2F2-null (E2F2 KO) mice and their wild-type (WT) littermates, VE-Cre; E2F3^+/+^ and E2F2 WT, respectively. The cardiac function, infarct size, and vascular density were significantly better in E2F2 KO mice and significantly worse in VE-Cre; E2F3^fl/fl^ mice than in their WT littermates. The loss of E2F2 expression was associated with an increase in the proliferation of ECs both in vivo and in vitro, while the loss of E2F3 expression led to declines in EC proliferation. Thus, E2F3 promotes while E2F2 suppresses ischemic cardiac repair through corresponding changes in EC proliferation; and differential targeting of specific E2F members may provide a novel strategy for therapeutic angiogenesis of ischemic heart disease.

## Introduction

Ischemic heart disease (IHD) represents one of the largest epidemics facing the aging population. Insufficient neovascularization, characterized by poor vessel growth and survival, contributes to the pathogenesis of IHD and limits cardiac tissue preservation and regeneration; thus enhancement of cardiac neovascularization is a therapeutic goal.

Following tissue ischemia, vascular injury recruits endothelial cells (ECs) required for neovascularization to restore blood perfusion [1]–[5]. Neovascularization is a complex sequence of events involving coordinated vascular cell proliferation, migration and tube formation [6]–[10]. Accumulating evidence indicates that neovascularization is critically dependent on the appropriate regulation of EC cell cycle [11], [12]; a deficient endothelial proliferative response to ischemia can result in tissues necrosis [13]. Additionally, ECs can contribute to cardiac repair by mediating favorable cell:cell interactions and secreting paracrine factors that protect the function and survival of cardiomyocytes [14].

The E2F family of transcription factors, with eight members identified, play a central role in regulating the expression of genes responsible for cell-cycle control and provide an ideal target for therapeutic modulation of vascular growth [15]–[20]. However, the PREVENT trial, targeting E2F activity in the vascular smooth muscle cells in the autologous vein grafts of CABG surgery to prevent graft overgrowth and failure, generated negative results presumably due to the non-specific inhibition of both activating and repressive E2F species [21], [22]; therefore, it is imperative to elucidate the specific function of individual E2F members in the vascular biology [20]. The E2F1, 2 and 3, in particular, are considered “transactivators” that activate transcription of target genes for DNA replication and G1/S transition, thus cell proliferation [23]–[26]. However, we have recently found that E2F1 *inhibits* ischemic angiogenesis by suppressing the expression of angiogenic factors, VEGF and PlGF [27]. The functions of E2F2 and E2F3 in ECs during ischemic disease are largely unknown.

In this study, we sought to investigate the roles of E2F2 and E2F3 in EC proliferation and their functional impact on myocardial infarction (MI). We found that E2F3 is essential for EC growth, neovascularization, and preservation of cardiac function, while E2F2 plays a contrasting role. Thus, individual E2F factors, rather than E2F family as a whole, may provide more specific molecular targets for therapeutic neovascularization of IHD.

## Materials and Methods

### Mice

The E2F3^fl/fl^ and E2F2^+/−^ mice were obtained from Dr. Gustavo Leone’s lab (Ohio State University) [23]. VE-cadherin-Cre (VE-Cre) mice were purchased from The Jackson Laboratory (Bar Harbor, ME). VE-Cre; E2F3^fl/fl^ mice and littermate VE-Cre; E2F3^+/+^ mice were generated by crossing VE-Cre; E2F3^fl/+^ male and E2F3^fl/+^ female parents. E2F2^−/−^ (E2F2 KO) and littermate E2F2^+/+^ (E2F2 WT) mice were generated by crossing E2F2^+/−^ parents as previously described [28]. All these mice were on C57BL/6 background. Mouse genotypes were determined via polymerase chain reaction (PCR) with tail DNA. All the animal work presented in this report was approved by the Institutional Animal Care and Use Committee of Northwestern University and performed in the barrier acilities of the Center for Comparative Medicine of the university.

### Surgical MI Model and Echocardiographic Assessments of Left Ventricular (LV) Function

MI was induced in 8 week-old male VE-Cre; E2F3^fl/fl^, VE-Cre; E2F3^+/+^, E2F2 KO, and E2F2 WT mice by permanent ligation in the middle of the left anterior descending (LAD) coronary artery as described previously [29], [30]. Mice were anesthetized by inhaling Isoflurane™ delivered at 2–4% throughout the surgical procedure, and were injected subcutaneously with Metacam (1 mg/kg) as analgesic immediately after the surgery and then daily for the next 2 to 3 days. Trans-thoracic 2-dimensional echocardiographic measurements were performed before MI (baseline) and at 7, 14 and 28 days post-MI by using a commercially available high resolution system (VEVO 770™, VisualSonics Inc., Toronto, Canada) equipped with a 30-MHz transducer. M-mode tracings were used to measure LV wall thickness, end-systolic diameter (LVESD), and end diastolic diameter (LVEDD). Systolic and diastolic LV areas were determined by M-mode in long-axis configuration and fractional shortening (FS) was measured at the mid-ventricular level. The LV chamber volumes in diastole and systole were derived from their respective measured 2D areas using a LV volume algorithm within the Vevo770 echo software. Cardiac ejection fraction (EF) was determined offline by the equation: EF = (Diastolic Volume - Systolic Volume/Diastolic Volume) x100.

### Histological Assessments of Infarct Size, Vascular Density, and *in vivo* EC Proliferation

Fourteen and 28 days after surgically induced MI, the vasculature was labeled by injecting 50 µL BS Lectin I (Vector laboratories, Inc., Burlingame, CA) into the tail vein, and mice were euthanized 10 min later by CO_2_ inhalation (primary method) and cervical dislocation (secondary method). A portion of animals also received Bromodeoxyuridine (BrdU) (30 mg/kg IP) for 48 h (Q12H×4) before euthanasia, which permits identification of proliferating cells. Cardiac tissues were fixed in 4% paraformaldehyde for 4 h, incubated overnight in 30% sucrose, embedded in OCT compound (Sakura Finetek U.S.A., Inc., Torrance, CA), snap-frozen in liquid nitrogen, and cut into 5 µm sections. Serial cryosectioning was performed starting at 1 mm below the suture (used to ligate the LAD) moving toward the apex, with three consecutive sections per 1 mm to allow for quantitative pathohistological analysis at each level. To evaluate infarct area, the Masson Trichrome elastic tissue staining was performed as described previously [30], [31]. Infarct size was reported as the ratio of the length of fibrotic area to the length of the LV inner circumference. Immunohistochemical staining was performed with fluorescent anti- BS Lectin 1 (Vector Laboratories, Inc.), anti-CD31 (Santa Cruz), and anti-BrdU (Abcam, Cambridge, MA, USA) antibodies to evaluate vascular density and proliferating cells as described previously [27], [29], [30]; 3 sections per ischemic heart and 6 fields per section were examined.

### Isolation of Mouse Primary Cardiac ECs and Adenoviral Vector Transduction

Primary ECs were isolated from mouse heart tissues as described previously [32]. Briefly, tissues were minced and digested with collagenase and dispase, then the mixture was passed through a 100-µm cell strainer to obtain single cell suspensions. Cell debris were removed via density centrifugation with Histopaque-1.083 (Sigma), then the ECs were immunostained with CD31-PE, FACS sorted to ≥95% purity, and cultured in EBM-2 medium (Lonza, Walkersville, MD). The cells were used before passage 5. The ECs were infected by adenovirus-Cre (Vector BioLabs, Philadelphia, PA) by following manufacturer’s instructions.

### EdU Incorporation Assay

Proliferation of primary ECs was measured with a commercially available Click-iT™ EdU (5-ethynyl-2′-deoxyuridine) Flow Cytometry Assay kit (Invitrogen) by following the manufacturer’s instructions. Prior to the addition of EdU, subconfluent ECs were synchronized by incubation in EBM-2 medium with 0.1% FBS for 24 h and stimulated to proliferate by addition of EBM-2 supplemented with 5% FBS.

### Statistical Analysis

All values are expressed as mean ± SEM. Comparison between two means was performed with an unpaired Student’s *t* test, whereas ANOVA with Fisher’s protected least significant differences and Bonferroni–Dunn post hoc analysis were used for comparisons of more than two means.

## Results

### Functional Recovery of the Infarcted Heart is Enhanced by the Loss of E2F2 Expression and Impaired by the Loss of Endothelial E2F3 Expression

**Figure 1:**
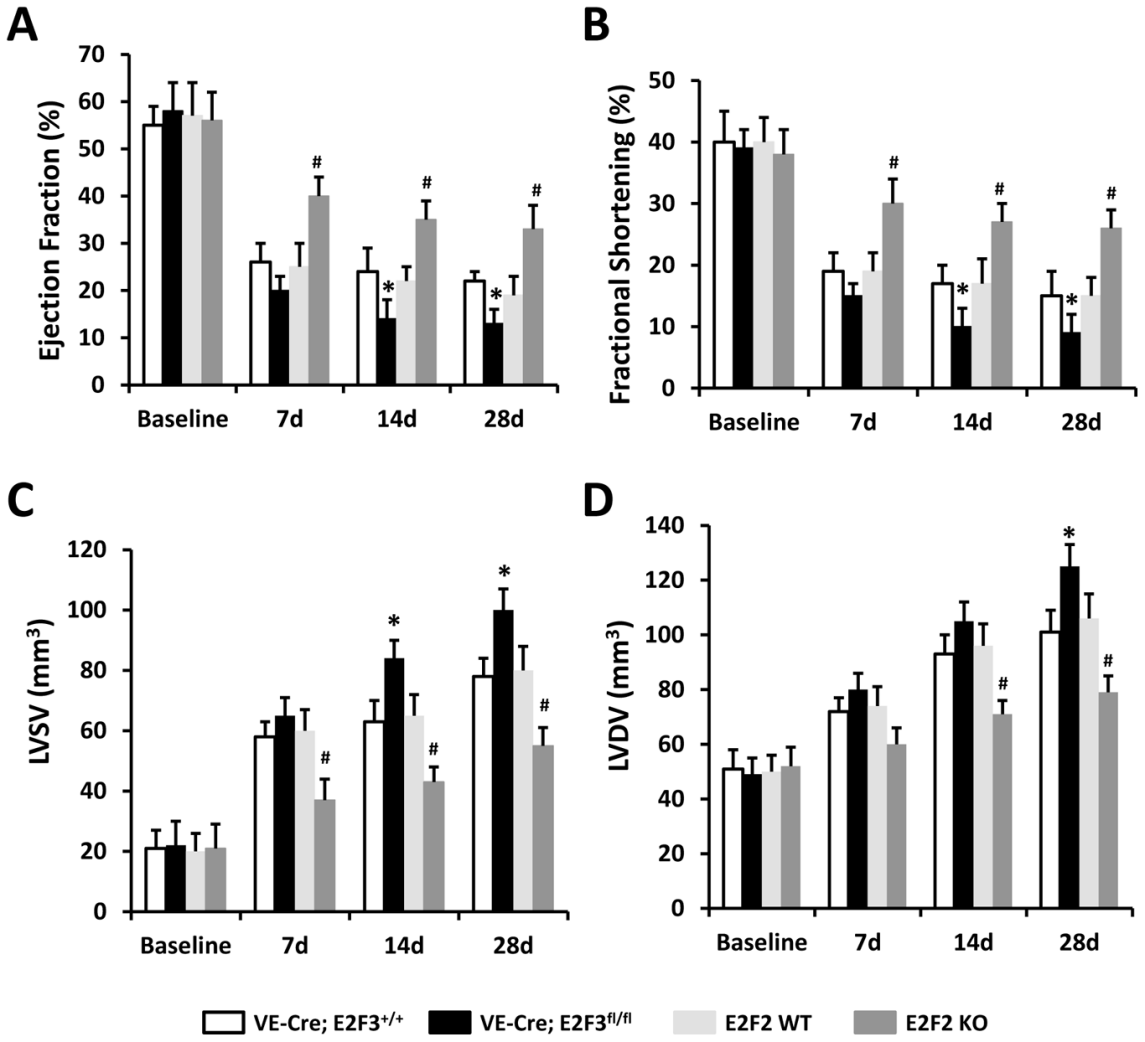
Functional recovery of the infarcted heart is enhanced by the loss of E2F2 expression and impaired by the loss of endothelial E2F3 expression. MI was surgically induced in VE-Cre; E2F3^fl/fl^ and E2F2 KO mice and their WT littermates, VE-Cre; E2F3^+/+^ and E2F2 WT, respectively, and the heart function was assessed with echocardiography at the indicated time points for (**A**) LV ejection fraction, (**B**) fractional shortening, (**C**) end-systolic and (**D**) end-diastolic volumes. n = 12 mice per group. *P<0.05 vs. VE-Cre; E2F3^+/+^, ^#^P<0.05 vs. E2F2 WT.

We induced MI by surgical ligation of LAD coronary artery in VE-Cre; E2F3^fl/fl^ and E2F2 KO mice and their WT littermates, VE-Cre; E2F3^+/+^ and E2F2 WT mice, respectively. E2F2 KO mice exhibited a greater EF and FS and a smaller LV systolic and diastolic volume as compared with E2F2 WT mice (Figure 1)**.** The significantly better cardiac function in E2F2 KO mice was observed as early as day 7 and persisted till day 28 post-MI. In contrast, VE-Cre; E2F3^fl/fl^ exhibited a worsened heart function as compared with VE-Cre; E2F3^+/+^ mice at days 14 and 28 post-MI (Figure 1). These results suggest that E2F3 improved while E2F2 impairs cardiac function in response to ischemic injury.

### Infarct Size and Vessel Density are Improved by the Loss of E2F2 Expression and Worsened by the Loss of Endothelial E2F3 Expression

**Figure 2:**
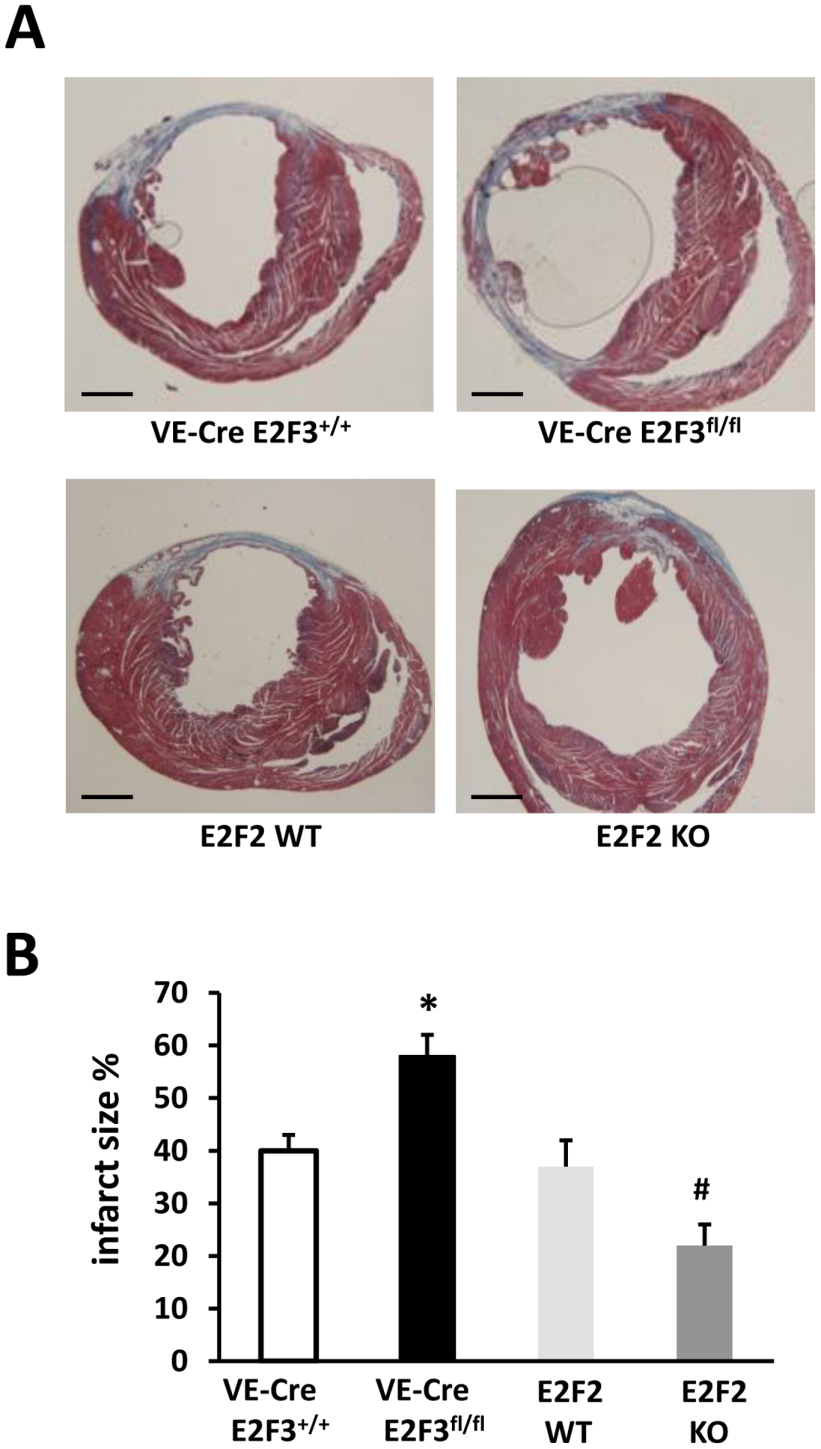
The infarct size is smaller in E2F2 KO mice and larger in endothelial specific E2F3 KO mice than in their WT littermates. Masson Trichrome staining was performed in heart samples 28 days after MI surgery. (**A**) Representative microphotographs and (**B**) Quantification of the infarct size. n = 12 mice per group; *P<0.05 versus VE-Cre; E2F3^+/+^, ^#^P<0.05 versus E2F2 WT; Scale bar = 100 µm.

**Figure 3:**
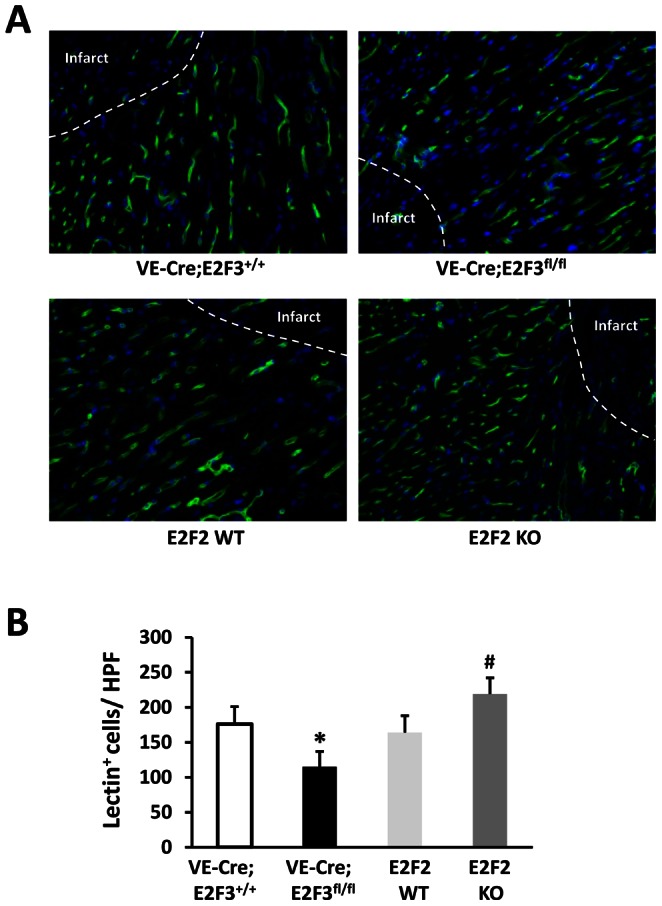
The vascular density at infarct border zone is greater in E2F2 KO mice and lower in endothelial E2F3 KO mice than in their WT littermates. Blood vessels were stained with BS lectin 1 (green), and nuclei were counterstained with DAPI (blue). (**A**) Representative immunofluorescence images. (**B**) Quantification of vascular density at the infarct border zone. n = 12 mice per group; *P<0.05 vs. VE-Cre; E2F3^+/+^, ^#^P<0.05 vs. E2F2 WT; HPF, high power field.

At day 28 post-MI, infarct size was significantly smaller in E2F2 KO and significantly larger in VE-Cre; E2F3^fl/fl^ mice, than in their littermates with WT levels of E2F2 and endothelial E2F3 expression (Figure 2). The loss of E2F2 expression in E2F2 KO mice also led to improvements in vessel density, while the endothelial deletion of E2F3 in VE-Cre; E2F3^fl/fl^ mice was associated with a decline in vessel density (Figure 3). Thus, E2F3 enhance while E2F2 suppresses cardiac neovascularization.

### EC Proliferation is Increased by Declines in E2F2 Expression and Reduced by Declines in E2F3 Expression

**Figure 4:**
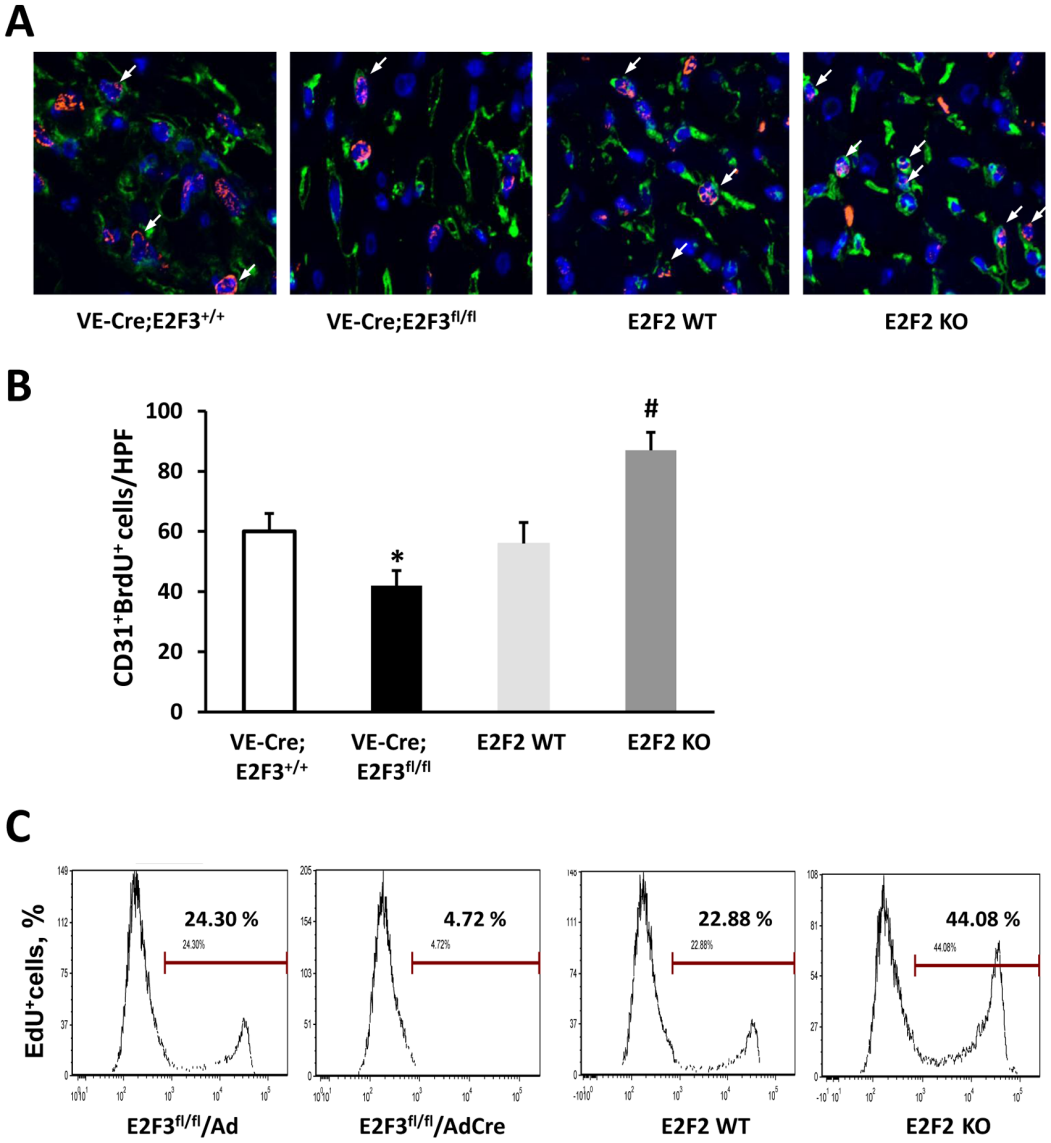
Proliferation is enhanced in E2F2 KO ECs and impaired in E2F3-deleted ECs. **(A–B)** Immunofluorescent double staining was performed in the ischemic heart sections for CD31 (green) and BrdU (red) to identify ECs (green), proliferating cells (red), and proliferating ECs (yellow). (**A**) Representative immunofluorescence images and (**B**) quantification of proliferating ECs in the infarct border zone. n = 12 mice per group; *P<0.05 vs. VE-Cre; E2F3^+/+^, ^#^P<0.05 vs. E2F2 WT; HPF, high power field. (**C**) Primary ECs were isolated from the hearts of E2F2 KO, E2F2 WT, and E2F3^fl/fl^ mice, and the E2F3^fl/fl^ cells were subsequently transduced with Adenovirus-Cre/GFP or Adenovirus-GFP. EdU incorporation based flow cytometry analyses were performed to assess DNA synthesis. Shown is representative of 3 independent experiments.

Because E2F2 and E2F3 are cell cycle regulators, we assessed EC proliferation in the ischemic tissue (i.e., the infarct border zone) at day 14 post-MI with CD31 and BrdU double staining. The frequency of proliferating ECs was significantly higher in E2F2 KO mice and significant lower in VE-Cre; E2F3^fl/fl^ mice, than in their WT littermates (Figure 4A–B).

To determine whether the contrasting roles of E2F2 and E2F3 in EC proliferation in vivo are cell autonomous, we isolated primary EC from the hearts of E2F2 KO, E2F2 WT, and E2F3^fl/fl^ mice and the E2F3^fl/fl^ ECs were subsequently transduced with a vector coding for GFP alone or with a vector coding for both GFP and Cre expression to knockout the expression of E2F3. Cells were starved to quiescence by serum deprivation and then re-stimulated by the addition of serum. The EdU incorporation assays showed that the proliferating (i.e., EdU+) ECs were more frequent in the E2F2 KO population and much less frequent in the E2F3 KO population, than in cells with cells with WT levels of E2F2 and E2F3 expression (Figure 4C). These results indicate that E2F2 suppresses and E2F3 enhances EC proliferation.

## Discussion

E2F1, 2, and 3 are classified as a subgroup of “activating” E2Fs that promote cell proliferation [33]–[37]. However, here we found that EC growth, neovascularization, and cardiac function post-MI are improved by the loss of E2F2 expression and impaired by the loss of endothelial E2F3 expression, which suggest that E2F2 and E2F3 play contrasting roles. Data from this current study and the results we have recently reported on E2F1 [27], collectively, suggest diverse but specific roles of these E2F species in the regulation of vascular growth.

Our data clearly show that E2F3 is essential for EC proliferation and ischemic angiogenesis, which cannot be compensated by other “activating” E2Fs (i.e., E2F1 and 2). Although consistent with reports in the literature documenting a critical role of E2F3 for cell cycle progression, this role appears to be greater in ECs than in other cell types [25], [38], [39]. Whether this role of E2F3 is specific to ECs or E2F3 plays a similar role in other vascular cells such as VSMCs remains to be investigated.

The finding that E2F2 suppresses EC proliferation is somewhat unexpected. It is in contrast to the observations made in several other cell types, in which overexpression of E2F2 activates cell cycle progression [40]. While the molecular mechanism underlying the enhanced growth of E2F2 KO ECs is yet to be determined, other lab did report that E2F2 KO mice display hyper-proliferation of immune cells [41]. Given the strong transactivity of E2F3 in ECs, it is tempting to speculate that in the absence of E2F2 (i.e., in E2F2 KO cells), the regulatory DNA elements normally bound by E2F2 may be occupied by E2F3 instead, thereby exerting a stronger transactivity for the expression of these genes. This hypothesis, however, remains to be tested in our future study.

In summary, our study revealed that E2F2 and E2F3, two members in the “activating” E2F subfamily exert contrasting roles in the regulation of EC growth and neovascularization and in the preservation of cardiac tissues from ischemic injury. Thus, individual E2Fs, rather than E2F family as a whole, may provide more specific targets for therapeutic angiogenesis of IHD.

## References

[1] Adams RH, Alitalo K (2007) Molecular regulation of angiogenesis and lymphangiogenesis. Nat Rev Mol Cell Biol 8: 464–478. 17522591 10.1038/nrm2183

[2] Carmeliet P (2005) Angiogenesis in life, disease and medicine. Nature 438: 932–936. 16355210 10.1038/nature04478

[3] Simons M (2005) Angiogenesis: where do we stand now? Circulation 111: 1556–1566. 15795364 10.1161/01.CIR.0000159345.00591.8F

[4] Robich MP, Chu LM, Oyamada S, Sodha NR, Sellke FW (2011) Myocardial therapeutic angiogenesis: a review of the state of development and future obstacles. Expert Review of Cardiovascular Therapy 9: 1469–1479. 22059795 10.1586/erc.11.148 PMC4839193

[5] Potente M, Gerhardt H, Carmeliet P (2011) Basic and Therapeutic Aspects of Angiogenesis. Cell 146: 873–887. 21925313 10.1016/j.cell.2011.08.039

[6] Chung AS, Ferrara N (2011) Developmental and Pathological Angiogenesis. Annual Review of Cell and Developmental Biology 27: 563–584. 10.1146/annurev-cellbio-092910-154002 21756109

[7] Fraisl P, Mazzone M, Schmidt T, Carmeliet P (2009) Regulation of Angiogenesis by Oxygen and Metabolism. Developmental Cell 16: 167–179. 19217420 10.1016/j.devcel.2009.01.003

[8] Weis SM, Cheresh DA (2011) Tumor angiogenesis: molecular pathways and therapeutic targets. Nat Med 17: 1359–1370. 22064426 10.1038/nm.2537

[9] Herbert SP, Stainier DYR (2011) Molecular control of endothelial cell behaviour during blood vessel morphogenesis. Nat Rev Mol Cell Biol 12: 551–564. 21860391 10.1038/nrm3176 PMC3319719

[10] Lähteenvuo J, Rosenzweig A (2012) Effects of Aging on Angiogenesis. Circulation Research 110: 1252–1264. 22539758 10.1161/CIRCRESAHA.111.246116 PMC4101916

[11] Semenza GL (2011) Hypoxia. Cross talk between oxygen sensing and the cell cycle machinery. American Journal of Physiology - Cell Physiology 301: C550–C552. 21677261 10.1152/ajpcell.00176.2011 PMC3174572

[12] Geudens I, Gerhardt H (2011) Coordinating cell behaviour during blood vessel formation. Development 138: 4569–4583. 21965610 10.1242/dev.062323

[13] Toutain CE, Brouchet L, Raymond-Letron I, Vicendo P, Bergès H, et al (2009) Prevention of Skin Flap Necrosis by Estradiol Involves Reperfusion of a Protected Vascular Network. Circulation Research 104: 245–254. 19059842 10.1161/CIRCRESAHA.108.182410

[14] Robich MP, Chu LM, Oyamada S, Sodha NR, Sellke FW (2011) Myocardial therapeutic angiogenesis: a review of the state of development and future obstacles. Expert Rev Cardiovasc Ther 9: 1469–1479. 22059795 10.1586/erc.11.148 PMC4839193

[15] Helin K (1998) Regulation of cell proliferation by the E2F transcription factors. Curr Opin Genet Dev 8: 28–35. 9529602 10.1016/s0959-437x(98)80058-0

[16] Dyson N (1998) The regulation of E2F by pRB-family proteins. Genes Dev: 2245–2262. 10.1101/gad.12.15.2245 9694791

[17] Wenzel PL, Chong J-L, Sáenz-Robles MT, Ferrey A, Hagan JP, et al (2011) Cell proliferation in the absence of E2F1-3. Developmental Biology 351: 35–45. 21185283 10.1016/j.ydbio.2010.12.025 PMC3868453

[18] Giangrande PH, Zhang J, Tanner A, Eckhart AD, Rempel RE, et al (2007) Distinct roles of E2F proteins in vascular smooth muscle cell proliferation and intimal hyperplasia. Proc Natl Acad Sci U S A 104: 12988–12993. 17652516 10.1073/pnas.0704754104 PMC1941807

[19] Spyridopoulos I, Principe N, Krasinski KL, Xu S, Kearney M, et al (1998) Restoration of E2F expression rescues vascular endothelial cells from tumor necrosis factor-alpha-induced apoptosis. Circulation 98: 2883–2890. 9860791 10.1161/01.cir.98.25.2883

[20] Qin G, Losordo DW (2008) E2F transcription factors in cardiovascular physiology. In: Yoshida K, editor. Control of Cellular Physiology by E2F Transciption Factors. Kerala: Research Signpost. 341–366.

[21] PREVENT IV Investigators (2005) Efficacy and Safety of Edifoligide, an E2F Transcription Factor Decoy, for Prevention of Vein Graft Failure Following Coronary Artery Bypass Graft Surgery: PREVENT IV: A Randomized Controlled Trial. JAMA 294: 2446–2454. 16287955 10.1001/jama.294.19.2446

[22] Alexander JH, Hafley G, Harrington RA, Peterson ED, Ferguson TB Jr, et al (2005) Efficacy and safety of edifoligide, an E2F transcription factor decoy, for prevention of vein graft failure following coronary artery bypass graft surgery: PREVENT IV: a randomized controlled trial. JAMA 294: 2446–2454. 16287955 10.1001/jama.294.19.2446

[23] Wu L, Timmers C, Maiti B, Saavedra HI, Sang L, et al (2001) The E2F1–3 transcription factors are essential for cellular proliferation. Nature 414: 457–462. 11719808 10.1038/35106593

[24] Nevins J (1998) Toward an understanding of the functional complexity of the E2F and retinoblastoma families. Cell Growth Differ 9: 585–593. 9716176

[25] Saavedra HI, Wu L, de Bruin A, Timmers C, Rosol TJ, et al (2002) Specificity of E2F1, E2F2, and E2F3 in Mediating Phenotypes Induced by Loss of Rb. Cell Growth Differ 13: 215–225. 12065245

[26] Chen H-Z, Tsai S-Y, Leone G (2009) Emerging roles of E2Fs in cancer: an exit from cell cycle control. Nat Rev Cancer 9: 785–797. 19851314 10.1038/nrc2696 PMC3616489

[27] Qin G, Kishore R, Dolan CM, Silver M, Wecker A, et al (2006) Cell cycle regulator E2F1 modulates angiogenesis via p53-dependent transcriptional control of VEGF. Proc Natl Acad Sci U S A 103: 11015–11020. 16835303 10.1073/pnas.0509533103 PMC1544166

[28] Zhou J, Zhu Y, Cheng M, Dinesh D, Thorne T, et al (2009) Regulation of vascular contractility and blood pressure by the E2F2 transcription factor. Circulation 120: 1213–1221. 19752322 10.1161/CIRCULATIONAHA.109.859207 PMC2785027

[29] Tang YL, Zhu W, Cheng M, Chen L, Zhang J, et al (2009) Hypoxic Preconditioning Enhances the Benefit of Cardiac Progenitor Cell Therapy for Treatment of Myocardial Infarction by Inducing CXCR4 Expression. Circ Res 104: 1209–1216. 19407239 10.1161/CIRCRESAHA.109.197723 PMC2756190

[30] Qin G, Ii M, Silver M, Wecker A, Bord E, et al (2006) Functional disruption of alpha4 integrin mobilizes bone marrow-derived endothelial progenitors and augments ischemic neovascularization. J Exp Med 203: 153–163. 16401693 10.1084/jem.20050459 PMC2118065

[31] Tang YL, Zhu W, Cheng M, Chen L, Zhang J, et al (2009) Hypoxic preconditioning enhances the benefit of cardiac progenitor cell therapy for treatment of myocardial infarction by inducing CXCR4 expression. Circ Res 104: 1209–1216. 19407239 10.1161/CIRCRESAHA.109.197723 PMC2756190

[32] Zhou J, Zhu Y, Cheng M, Dinesh D, Thorne T, et al (2009) Regulation of Vascular Contractility and Blood Pressure by the E2F2 Transcription Factor. Circulation 120: 1213–1221. 19752322 10.1161/CIRCULATIONAHA.109.859207 PMC2785027

[33] DeGregori J, Johnson D (2006) Distinct and Overlapping Roles for E2F Family Members in Transcription, Proliferation and Apoptosis Current Molecular Medicine. 6: 739–748. 10.2174/1566524010606070739 17100600

[34] Trimarchi JM, Lees JA (2002) Sibling rivalry in the E2F family. Nat Rev Mol Cell Biol 3: 11–20. 11823794 10.1038/nrm714

[35] Wu L, Timmers C, Maiti B, Saavedra HI, Sang L, et al (2001) The E2F1–3 transcription factors are essential for cellular proliferation. Nature 414: 457–462. 11719808 10.1038/35106593

[36] Helin K (1998) Regulation of cell proliferation by the E2F transcription factors. Current Opinion in Genetics & Development 8: 28–35. 9529602 10.1016/s0959-437x(98)80058-0

[37] Tao Y, Kassatly RF, Cress WD, Horowitz JM (1997) Subunit composition determines E2F DNA-binding site specificity. Molecular and Cellular Biology 17: 6994–7007. 9372931 10.1128/mcb.17.12.6994 PMC232556

[38] Field SJ, Tsai F-Y, Kuo F, Zubiaga AM, Kaelin Jr WG, et al (1996) E2F-1 Functions in Mice to Promote Apoptosis and Suppress Proliferation. Cell 85: 549–561. 8653790 10.1016/s0092-8674(00)81255-6

[39] DeGregori J (2002) The genetics of the E2F family of transcription factors: shared functions and unique roles. Biochimica et Biophysica Acta (BBA) - Reviews on Cancer 1602: 131–150. 12020800 10.1016/s0304-419x(02)00051-3

[40] Muller H, Bracken AP, Vernell R, Moroni MC, Christians F, et al (2001) E2Fs regulate the expression of genes involved in differentiation, development, proliferation, and apoptosis. Genes Dev 15: 267–285. 11159908 10.1101/gad.864201 PMC312619

[41] Murga M, Fernandez-Capetillo O, Field SJ, Moreno B, Borlado LR, et al (2001) Mutation of E2F2 in mice causes enhanced T lymphocyte proliferation, leading to the development of autoimmunity. Immunity 15: 959–970. 11754817 10.1016/s1074-7613(01)00254-0

